# Complete Genome Sequence of Pseudomonas amygdali pv. tabaci Strain 6605, a Causal Agent of Tobacco Wildfire Disease

**DOI:** 10.1128/MRA.00405-21

**Published:** 2021-07-15

**Authors:** Hidenori Matsui, Takafumi Nishimura, Shuta Asai, Sachiko Masuda, Ken Shirasu, Mikihiro Yamamoto, Yoshiteru Noutoshi, Kazuhiro Toyoda, Yuki Ichinose

**Affiliations:** a Graduate School of Environmental and Life Sciences, Okayama University, Okayama, Okayama, Japan; b Center for Sustainable Resource Science, RIKEN, Yokohama, Kanagawa, Japan; Loyola University Chicago

## Abstract

Pseudomonas amygdali pv. tabaci strain 6605 is the bacterial pathogen causing tobacco wildfire disease that has been used as a model for elucidating virulence mechanisms. Here, we present the complete genome sequence of P. amygdali pv. tabaci 6605 as a circular chromosome from reads using a PacBio sequencer.

## ANNOUNCEMENT

Pseudomonas species are phytopathogens responsible for plant diseases affecting many agricultural species (1). Pseudomonas amygdali pv. tabaci (former name: Pseudomonas syringae pv. tabaci) strain 6605, a Gram-negative bacterium responsible for tobacco wildfire disease, has been used as a model for studying the virulence mechanisms of plant pathogens (2, 3). About a decade ago, a draft genome sequence of P. amygdali pv. tabaci 6605 was released, contributing to the study of plant-microbe interactions (4, 5). However, the draft genome assembly, created using Illumina short reads, was too fragmented to elucidate the virulence of the pathogen. Here, we report the complete genome sequence of P. amygdali pv. tabaci 6605, characterized using PacBio sequencing.

*P. amygdali* pv. tabaci 6605 is a strain originally isolated in Nagasaki Prefecture, Japan. The stock culture was streaked onto King’s B (KB) agar plates. A single colony was cultured in 10 ml of KB liquid medium overnight at 27°C with shaking at 180 rpm. DNA was extracted using cetyltrimethylammonium bromide (CTAB) and Genomic-tip 100/G columns (Qiagen, Hilden, Germany), using a method archived by the 1000 Fungal Genomes Project (http://1000.fungalgenomes.org/home/wp-content/uploads/2013/02/genomicDNAProtocol-AK0511.pdf).

The genome of P. amygdali pv. tabaci 6605 was sequenced along with several other bacterial strains using the PacBio Sequel system (Menlo Park, CA, USA). PacBio libraries were prepared and size selected with a 20-kb cutoff using a Megaruptor 2 system (Diagenode, Seraing [Ougrée], Belgium), and the libraries were constructed using a SMRTbell template prep kit 1.0 according to the manufacturer’s protocol (Pacific Biosciences). Barcodes were attached to each fragmented genome, and the samples were pooled and cut off at 12 kbp using the BluePippin size selection system (Sage Science, Beverly, MA, USA). The genomic library was sequenced on a single PacBio Sequel system 1M v.3 cell. A total of 243,295,583 filtered subreads (*N*_50_, 15,015 bp) were assembled using the Hierarchical Genome Assembly Process (HGAP) v.4 within SMRT Link v.7.0.1, with the expected genome size set to 6 Mb. Circlator v.1.5.5 (6) was used to evaluate whether the assemblies were circular and to predict the location of the starting position.

**FIG 1:**
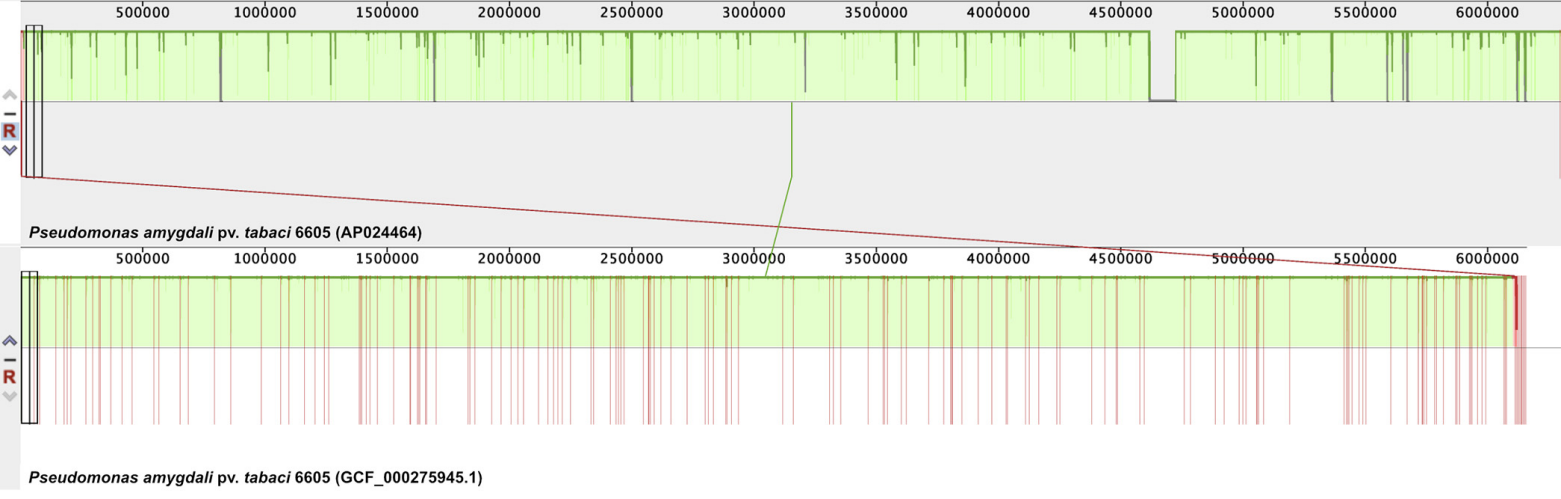
Alignment of the complete genome sequence (GenBank accession number AP024464) and the draft genome sequence (GCF_000275945.1) of *P. amygdali* pv. tabaci 6605 using the Mauve program. The contigs are separated by red lines.

The P. amygdali pv. tabaci 6605 genome sequence has a total length of 6,299,574 bp with an average GC content of 58.0% and an average sequence depth of coverage exceeding 86×, and it consists of one circular chromosome (6,299,574 bp). The genome was automatically annotated using the DFAST pipeline (7) to predict a total of 5,623 protein-coding sequences, 66 tRNAs, and 16 rRNA (5S, 16S, and 23S rRNA) operons. A comparative analysis using Mauve v.2.4.0 (8) showed close agreement with the previous draft P. amygdali pv. tabaci 6605 genome sequence (GenBank accession number GCF_000275945.1), complementing the gaps between contigs (Fig. 1). Default parameters were used for all software unless otherwise specified. The complete genome sequence of P. amygdali pv. tabaci 6605 will provide insights into the molecular evolution of phytopathogenic bacteria and their virulence mechanisms.

### Data availability.

This complete genome project has been deposited in DDBJ/ENA/GenBank under the accession number AP024464 (BioProject accession number PRJDB11119, BioSample accession number SAMD00276453, and DRA accession number DRA012177). The version described in this paper is the first version, AP024464.

## References

[1] Mansfield J, Genin S, Magori S, Citovsky V, Sriariyanum M, Ronald P, Dow M, Verdier V, Beer SV, Machado MA, Toth I, Salmond G, Foster GD. 2012. Top 10 plant pathogenic bacteria in molecular plant pathology. Mol Plant Pathol 13:614–629. doi:10.1111/j.1364-3703.2012.00804.x. 22672649 PMC6638704

[2] Taguchi F, Ichinose Y. 2011. Role of type IV pili in virulence of *Pseudomonas syringae* pv. *tabaci* 6605: correlation of motility, multidrug resistance, and HR-inducing activity on a nonhost plant. Mol Plant Microbe Interact 24:1001–1011. doi:10.1094/MPMI-02-11-0026. 21615203

[3] Buscaill P, Chandrasekar B, Sanguankiattichai N, Kourelis J, Kaschani F, Thomas EL, Morimoto K, Kaiser M, Preston GM, Ichinose Y, van der Hoorn RAL. 2019. Glycosidase and glycan polymorphism control hydrolytic release of immunogenic flagellin peptides. Science 364:eaav0748. 30975858 10.1126/science.aav0748

[4] Winsor GL, Griffiths EJ, Lo R, Dhillon BK, Shay JA, Brinkman FSL. 2016. Enhanced annotations and features for comparing thousands of *Pseudomonas* genomes in the *Pseudomonas* genome database. Nucleic Acids Res 44:D646–D653. doi:10.1093/nar/gkv1227. 26578582 PMC4702867

[5] Studholme DJ. 2011. Application of high-throughput genome sequencing to intrapathovar variation in *Pseudomonas syringae*. Mol Plant Pathol 12:829–838. doi:10.1111/j.1364-3703.2011.00713.x. 21726380 PMC6640474

[6] Hunt M, De Silva N, Otto TD, Parkhill J, Keane JA, Harris SR. 2015. Circlator: automated circularization of genome assemblies using long sequencing reads. Genome Biol 16:294. doi:10.1186/s13059-015-0849-0. 26714481 PMC4699355

[7] Tanizawa Y, Fujisawa T, Nakamura Y. 2018. DFAST: a flexible prokaryotic genome annotation pipeline for faster genome publication. Bioinformatics 34:1037–1039. doi:10.1093/bioinformatics/btx713. 29106469 PMC5860143

[8] Darling ACE, Mau B, Blattner FR, Perna NT. 2004. Mauve: multiple alignment of conserved genomic sequence with rearrangements. Genome Res 14:1394–1403. doi:10.1101/gr.2289704. 15231754 PMC442156

